# Unraveling the Enigma of Melanoma Brain Metastasis: New Molecular Insights and Therapeutic Directions

**DOI:** 10.3390/ijms27042019

**Published:** 2026-02-20

**Authors:** Kayla T. O’Toole, Brandon M. Roan, Timothy M. Hardman, Peyton P. Phillips, Evan M. VanBrocklin, Gennie L. Parkman, Sheri L. Holmen

**Affiliations:** 1 Department of Oncological Sciences, University of Utah Health Sciences Center, Salt Lake City, UT 84112, USA; kayla.otoole@hci.utah.edu; 2 Jerry M. Wallace School of Osteopathic Medicine, Campbell University, Lillington, NC 27507, USA; bmroan0701@email.campbell.edu (B.M.R.); t_hardman0726@email.campbell.edu (T.M.H.);; 3 Department of Surgery, University of Utah Health Sciences Center, Salt Lake City, UT 84112, USA; evan.vanbrocklin@hci.utah.edu; 4 Department of Zoology, Weber State University, Ogden, UT 84403, USA; gennieparkman@weber.edu; 5 Huntsman Cancer Institute, University of Utah Health Sciences Center, Salt Lake City, UT 84112, USA

**Keywords:** melanoma, brain metastases, NRAS, BRAF, PI3K/AKT, immune checkpoint inhibitors, brain microenvironment, preclinical models, therapeutic resistance

## Abstract

Melanoma, a highly aggressive and metastatic cancer, poses significant challenges due to its propensity to spread to distant organs, with brain metastasis representing a particularly devastating complication. This review synthesizes preclinical and clinical evidence on the molecular, cellular, and microenvironmental mechanisms driving melanoma metastasis, emphasizing mechanisms of blood–brain barrier traversal, tumor-stroma co-option, and brain-specific genomic and transcriptional programs. We summarize advances in therapeutic strategies to combat melanoma brain metastasis including novel small molecules, immunotherapies, and combination approaches tailored for brain metastases. The review also highlights the immunological landscape of the brain, translational models, and multidisciplinary clinical management strategies. Finally, we identify critical research gaps, including the need for brain metastasis-specific clinical trials, AI-driven predictive models, and preventive strategies, to guide future efforts in improving outcomes for patients with melanoma brain metastasis.

## 1. Introduction

Melanoma is an aggressive form of skin cancer and the 5th most common malignancy in the United States [1]. While frontline treatment strategies have greatly improved patient outcomes over the last decade, melanoma remains a deadly disease. Importantly, the majority of melanoma-related deaths result from distant metastases [1,2]. Incidence rates vary widely by region with fewer than 0.5 cases per 100,000 in Asia, approximately 1 per 100,000 in Africa, 13.2 per 100,000 in Europe, 21.6 per 100,000 in the United States, and the highest rates in Australia at 48 per 100,000 [3], contributing to a global annual increase of approximately 2.6%. In the United States, an estimated 8430 melanoma-related deaths occurred in 2025 [4]. Although melanoma incidence continues to increase, mortality rates began decreasing in 2013, largely due to the development of both targeted therapies and immunotherapies [5]. However, while the 5-year survival rate for patients with localized melanoma approaches 100% when restricted to stage I (stage II comprises the high-risk categories IIB and IIC), survival drops below 35% for patients with distant metastatic disease despite current treatments [6].

Melanoma arises from melanocytes, pigment-producing cells that synthesize melanin, which serves as a protective barrier against ultraviolet (UV) radiation. Melanocytes are derived from neural crest cells during embryonic development and reside in the basal layer of the epidermis [7]. Historically, melanoma progression has been described as a linear, stepwise process in which metastatic dissemination does not occur until late-stage disease progression, with depth of invasion defined by the Clark model staging system [8]. Critical steps in melanoma progression begin with the formation of benign melanocytic and dysplastic nevi, followed by primary tumor growth, and ultimately culminating in metastasis, driven by the accumulation of genetic alterations [9]. In this framework, metastasis is restricted to advanced stages of disease and is attributed to a limited subset of tumor clones with metastatic potential. However, accumulating evidence indicates that melanoma progression is frequently non-linear and biologically more complex than this traditional model suggests.

**Figure 1:**
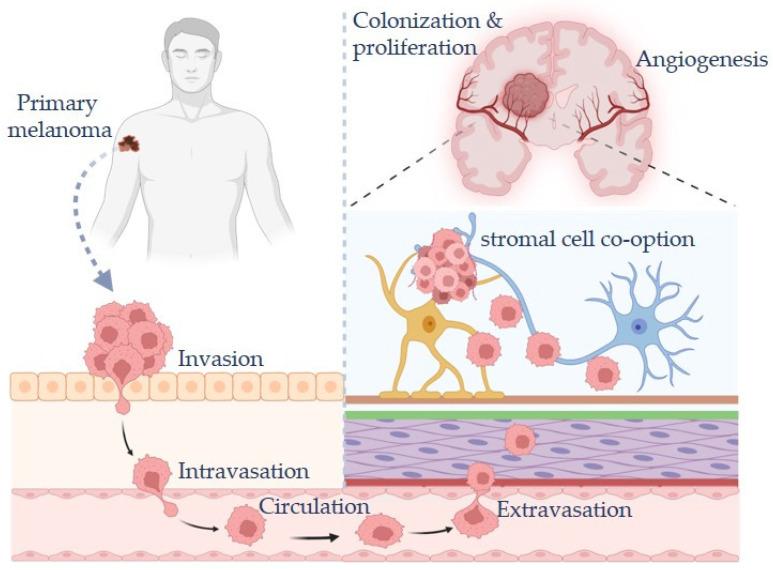
Steps involved in melanoma metastasis from the primary tumor to the brain. Figure created with BioRender.com.

Melanoma is characterized by a pronounced capacity for metastatic dissemination, driven by biological features that facilitate vascular invasion, immune evasion, and colonization of distant organs. Metastasis is initiated when malignant cells from superficial primary lesions invade the deeper dermal layers, enabling entry into the vascular and lymphatic compartments. Dissemination of melanoma cells to the brain is a complex, multistep process that begins with intravasation into lymphatic or blood vessels. Circulating tumor cells must survive the hostile conditions of the circulation, express adhesion molecules that mediate interactions with vascular endothelium, and ultimately extravasate across the BBB [10,11,12]. This process is particularly challenging due to the presence of tight-junction adhesions and low permeability within the brain endothelium [13]. Following intravasation, melanoma cells spread to regional and distant sites via lymphatic and hematogenous circulation [14]. Melanoma cells also acquire endothelial-like properties that enhance survival within the circulation and facilitate adhesion and extravasation at secondary sites (Figure 1). In parallel, the expression of pro-inflammatory and pro-angiogenic mediators supports tumor vascularization and metastatic outgrowth [15,16,17].

Metastatic progression is the primary determinant of melanoma prognosis. Notably, advanced nodular lesions may be metastatic at initial staging; conversely, metastatic lesions may sometimes have an occult primary tumor, limiting the effectiveness of surgical resection alone and necessitating aggressive multimodal treatment strategies, as removal of the primary lesion does not reliably prevent further spread [1]. Among metastatic sites, melanoma exhibits a particularly high propensity for brain colonization, which represents a major source of morbidity and mortality. An estimated 40–75% of patients with stage IV disease develop intracranial metastases, which is associated with a median survival of ~9 months from the time of brain metastasis diagnosis in the modern treatment era [18].

Although metastatic melanoma has historically been associated with poor outcomes, recent advances in immune checkpoint inhibition and targeted therapies have begun to improve survival, including in patients with brain involvement [19,20,21,22,23,24,25]. This review summarizes current insights into the mechanisms underlying melanoma metastasis, with a particular emphasis on brain colonization, and highlights emerging therapeutic strategies and future research directions.

## 2. Mechanisms of Melanoma Metastasis

Cutaneous melanoma is distinguished by an exceptionally high mutational burden and intratumoral heterogeneity, reflecting the involvement of numerous genetic drivers in disease initiation and progression. This extensive genetic complexity poses substantial challenges for therapeutic targeting and contributes to the rapid emergence of treatment resistance [26,27,28,29]. Notably, melanoma recurrence rarely occurs at the primary tumor site and instead frequently presents as metastatic lesions at distant sites [30], suggesting that dissemination likely occurs prior to surgical removal of the primary lesion.

**Figure 2:**
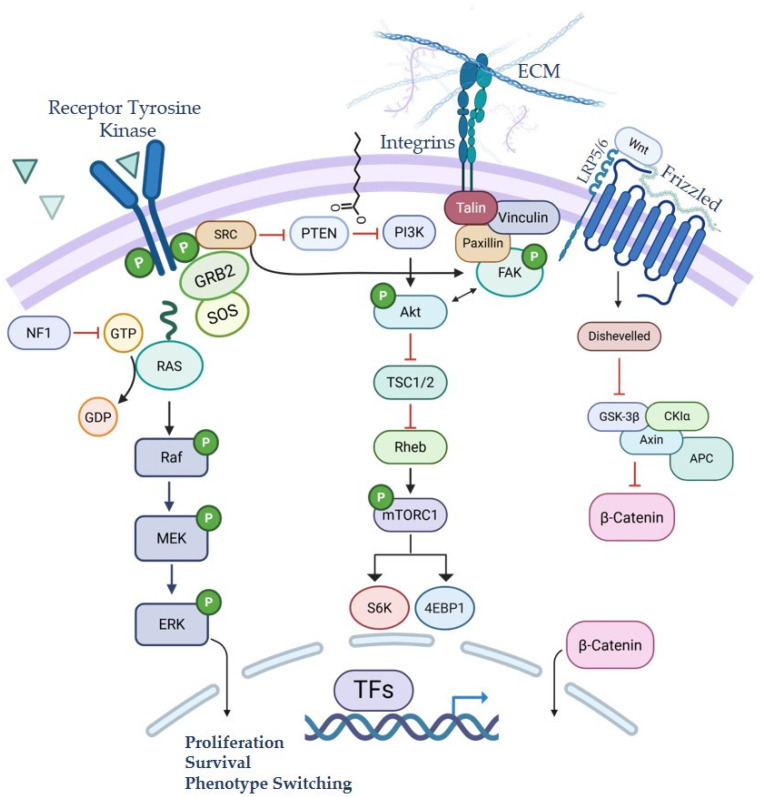
Signaling pathways altered in melanoma with an emphasis on those that drive metastasis to the brain. TFs: transcription factors; ECM: extracellular matrix. Figure created with BioRender.com.

The likelihood of melanoma metastasis is influenced by multiple factors, ranging from tumor microenvironment cues to intrinsic changes in cell state and differentiation [31]. Activation of diverse oncogenic cell signaling pathways is a hallmark of melanoma development, progression, and metastasis. Among these, aberrant activation of the mitogen-activated protein kinase (MAPK) pathway occurs in the majority of melanoma cases, most commonly through oncogenic mutations in *BRAF*, which are present in over 50% of all cutaneous melanoma cases and represent the most frequently altered oncogene in this malignancy (Figure 2). The most common oncogenic alteration, *BRAF^T1799A^*, encodes the BRAF^V600E^ oncoprotein kinase which induces a strong gain-of-function phenotype and leads to constitutive BRAF signaling with sustained ERK activation.

Clinically, BRAF-mutant melanomas exhibit an increased propensity for brain metastasis [32,33], with specific mutational subtypes (e.g., V600K) associated with distinct survival outcomes [34]. Beyond proliferative signaling, BRAF alterations have been implicated in melanogenesis, phenotypic plasticity, and immune modulation within the tumor microenvironment, collectively facilitating metastatic spread [32]. Consequently, selective mutant BRAF inhibitors, in combination with MEK inhibitors, have become a cornerstone of targeted therapy for patients with BRAF-mutant melanoma [35].

*NRAS*, which encodes a membrane-associated GTPase in the RAS family upstream of the RAF family kinases, is mutated in approximately 20% of cutaneous melanomas and was among the earliest oncogenic drivers identified in this disease [36]. NRAS transduces receptor-mediated signals through the Ras-regulated RAF—MEK—ERK MAPK cascade, leading to ERK-mediated phosphorylation of numerous cytoplasmic and nuclear targets, including the lineage-defining transcription factor MITF [29,37,38] (Figure 2). Although targeting RAS has historically proven challenging in numerous solid tumor types, earning its reputation as “undruggable”, recent advancements in targeted therapies have begun to show promise. This is further discussed in Section 7.

Activation of RAS isoforms (H-, N-, or K-) also triggers downstream activation of the phosphatidylinositol 3-kinase (PI3K) pathway, another critical driver of melanoma progression. PI3K activation leads to downstream phosphorylation of the serine/threonine kinase AKT, which promotes cell survival through inhibition of pro-apoptotic mediators such as Bad. In melanoma, PI3K pathway activation is associated with enhanced tumor growth, angiogenesis, metastatic dissemination, and therapeutic resistance [39] (Figure 2).

Importantly, in some contexts silencing of *PTEN* or activation of PI3K (H1047R) has minimal effects on normal melanocytes, indicating that these alterations likely do not drive melanoma initiation but promote its disease progression [40]. While it is well-established that melanomagenesis requires numerous cooperating events beyond activation of common drivers such as *BRAF* or *NRAS*, melanoma metastasis likewise depends on additional genetic events, the majority of which converge on activation of PI3K lipid signaling [41,42]. Specifically, loss of *PTEN* has been shown to cooperate with the BRAF^V600E^ mutation to drive metastatic melanoma [43]. In parallel, activation of AKT can independently promote melanoma metastasis to the lung and brain, even in the absence of PTEN inactivation [44,45,46,47,48]. Notably, melanomas with brain metastases frequently exhibit upregulated PI3K signaling (Figure 2), and although pharmacologic inhibition of this pathway can suppress tumor growth in preclinical and early clinical settings, therapeutic efficacy has been limited by dose-limiting toxicities and the requirement for simultaneous inhibition of multiple paralogs in the pathway [49,50,51,52].

### 2.1. Epithelial-to-Mesenchymal Transition [EMT] and Plasticity in Melanoma

Epithelial-to-mesenchymal transition (EMT) is a key driver of metastasis in many solid tumors. Although not epithelial in origin, melanoma cells can exhibit an EMT-like transition that shares molecular parallels with classical EMT in epithelial cancers, involving transcription factors such as ZEB1, TWIST1, and TCF4 that repress differentiation markers and facilitate metastatic propensity. This EMT-like transition is particularly relevant to central nervous system (CNS) dissemination, as melanoma brain metastases exhibits upregulation of EMT-associated genes that facilitate motility, vascular transit, blood–brain barrier (BBB) traversal, and adaptation to the cerebral microenvironment during invasion and colonization [53].

A central regulator of melanocyte biology and melanoma behavior is the lineage-specific transcription factor microphthalmia-associated transcription factor (MITF). In melanoma, MITF operates according to a “rheostat” model, whereby low MITF levels are associated with an invasive, therapy-resistant state, while higher levels promote proliferation and differentiation [54,55,56,57]. MITF expression is tightly regulated by upstream signaling pathways, including MAPK and canonical Wnt/β-catenin signaling, as well as by the transcription factor SOX10, all of which contribute to melanoma progression and immune evasion [58,59].

Oncogenic BRAF and NRAS signaling further reinforce EMT-like programs through transcriptional switching from ZEB2/SNAIL2 to ZEB1/TWIST1, accompanied by reduced E-cadherin expression and increased mesenchymal markers such as N-cadherin, promoting invasiveness and metastatic potential [60,61]. Additional regulators, including SPARC and PRRX1, strengthen these programs via activation of β-catenin and TGF-β signaling pathways [16,62,63]. Collectively, EMT-like phenotypic plasticity enables melanoma cells to disseminate and successfully colonize the brain.

### 2.2. Tumor Microenvironment Contributions to Metastasis

The tumor microenvironment (TME) surrounding melanoma brain metastasis plays a central role in immune evasion, therapeutic resistance, as well as metastatic outgrowth, and has therefore emerged as a critical focus of clinical and translational research. The melanoma brain metastatic TME is highly heterogenous, comprising immune and stromal cell populations, extracellular matrix (ECM) components, and a wide array of soluble factors including cytokines, chemokines, and growth factors. A major focus of TME investigation has centered on the immune landscape within this niche and the mechanisms of melanoma cell immune evasion.

A principal mechanism of immune escape involves activation of inhibitory immune checkpoints that suppress cytotoxic T lymphocyte (CTL) function. Within the tumor microenvironment, persistent antigen exposure and immunosuppressive signaling are associated with upregulation of CTLA-4 and PD-1 on CTLs, resulting in impaired T-cell activation and functional exhaustion [64]. These pathways will be discussed further below as they are therapeutically targeted by immune checkpoint inhibitors (ICIs) [65,66,67,68,69].

Beyond T cells, innate and adaptive immune populations also contribute to immune evasion in melanoma brain metastasis. Neutrophils have emerged as key mediators of therapeutic resistance, with non-responding intracranial tumors exhibiting neutrophil-rich microenvironments despite comparable levels of T-cell infiltration [70]. In particular, IL-8–expressing neutrophil subpopulations, along with those producing interferons and calprotectin, promote angiogenesis, aggressive tumor growth, and EMT, suggesting that neutrophil-driven inflammation contributes to immune escape and metastatic persistence within the central nervous system [71].

B-cells also represent a prominent immunomodulatory population in melanoma brain metastases, occurring at higher frequencies than in brain metastases derived from breast or lung cancers [72]. Regulatory B-cells secrete anti-inflammatory cytokines such as IL-10, which suppress CTL activity and impair antigen presentation, thereby attenuating antitumor immune responses [73].

Stromal cells further shape the immunosuppressive and pro-metastatic niche of melanoma brain metastases. Although normally essential for maintaining BBB integrity and immune homeostasis, stromal cells can undergo tumor-induced reprogramming that supports metastatic growth, inflammation, and immune evasion [74]. Activated stromal signaling pathways, including YAP1 downstream of β-catenin, promote EMT-like transition through induction of a ZEB2-to-ZEB1 transcriptional switch in adjacent melanoma cells and may also drive tumor vasculogenesis [75,76,77]. Age-associated fibroblast changes further exacerbate metastatic progression, including secretion of the Wnt antagonist sFRP2, which suppresses β-catenin, MITF, and APE1, thereby promoting angiogenesis, metastasis, and resistance to BRAF-targeted therapies [25].

Remodeling of the ECM is another defining feature of melanoma brain metastasis. Loss of basement membrane integrity, particularly through collagen IV degradation, facilitates tumor invasion, while increased matrix stiffness promotes hypoxia, focal adhesion signaling, drug resistance, and metastatic fitness [78,79]. Aged fibroblasts contribute to ECM remodeling by increasing endothelial permeability, impairing lymphatic function, and promoting visceral metastasis. These fibroblasts also secrete ceramide lipids that are taken up by melanoma cells via fatty acid transport protein 2 (FATP2), encoded by the SLC27A2 gene, supporting resistance to oxidative stress and targeted therapies such as BRAF/MEK inhibitors [80,81]. Melanoma cells also actively remodel their metastatic niche through intercellular communication. The secretion of nerve growth factor receptor (NGFR)-containing extracellular vesicles enhances lymphangiogenesis and tumor-endothelial adhesion, thereby facilitating metastatic dissemination and colonization of the brain [82].

Integration of multi-omics approaches, spanning genomics, transcriptomics, proteomics, metabolomics, and epigenomics, has substantially advanced our understanding of the molecular determinants that enable melanoma cells to thrive and spread in the brain. Recent studies reveal that melanoma brain metastases harbor distinct molecular programs that drive CNS tropism, immune escape, metabolic reprogramming, and therapeutic resistance [83]. Single-cell multi-omic analyses have identified a pre-metastatic melanoma subpopulation with upregulated neural adhesion genes (e.g., *NRG3*, *NCAM1*) that predisposes tumors to brain colonization [83,84,85]. Complementary genomic and transcriptomic profiling reveals that melanoma brain metastases frequently harbor mutations such as loss-of-function *PTEN* and display brain-like gene expression signatures not observed in extracranial lesions, underscoring the importance of context-specific modeling platforms [83,84,85].

## 3. Translational and Preclinical Models for Studying Brain Metastasis

The development and application of experimental models has been essential for advancing our understanding of melanoma biology, including early oncogenic events, metastatic progression, immune interactions, and therapeutic resistance [86]. Current approaches encompass a broad spectrum of in vitro and in vivo systems including tumor-derived cell lines, patient-derived xenografts (PDX), three-dimensional (3D) cultures, such as organoids, zebrafish, and genetically-engineered mouse models (GEMMs) [86].

Tumor-derived melanoma cell lines remain widely used due to their reproducibility, suitability for high-throughput experimentation, and ease of genetic manipulation. While these systems enable mechanistic interrogation and drug discovery efforts, they are inherently limited by selection bias toward culture-adapted clones, absence of a physiologically relevant microenvironment, and lack of critical stromal, vascular, and immune components that influence tumor behavior in vivo [87,88]. PDX models partially address these shortcomings by engrafting human tumor tissue into immunocompromised mice, thereby preserving intratumoral heterogeneity and enabling assessment of therapeutic responses across architectural, histological, phenotypic, and mutational dimensions. However, dependence on murine host stroma and lack of a fully functioning immune system limits the capability of PDX models to faithfully recapitulate human tumor-microenvironment interactions [86,89].

Advances in 3D melanoma modeling have yielded platforms that more closely mimic native tissue architecture and tumor microenvironments. Spheroid cultures, composed of aggregated melanoma cells grown in suspension, develop oxygen and nutrient gradients that generate intratumoral heterogeneity and enhance physiological relevance relative to two-dimensional cultures [90,91]. When co-cultured with additional cell types, spheroids can provide insight into early steps of the metastatic cascade; nevertheless, their capacity to model complex host–tumor interactions remain limited [92,93]. Organoid models represent a further evolution of 3D systems, incorporating layered skin cell populations within supportive scaffolds. Recent bioengineering innovations have enabled the inclusion of appendages such as hair follicles, sebaceous and sweat glands, and vascular-like structures, reducing necrosis and enhancing tissue fidelity [94]. Importantly, patient-derived melanoma organoids generated from metastatic lesions have recently been shown to be feasible, enabling personalized modeling of disease progression and therapeutic response [95,96,97]. Skin reconstructs employ primary human keratinocytes, melanocytes, and fibroblasts embedded within extracellular matrix components to generate stratified epidermal structures that closely resemble native human skin. These platforms enable detailed investigation of cell–cell interactions, stromal influences, and melanoma initiation and progression within a customizable microenvironment [98,99].

Mouse models have long formed the foundation of preclinical melanoma research and have played a central role in the development of effective therapeutic strategies. Syngeneic mouse models of melanoma brain metastasis primarily utilize immunocompentent C57BL/6 mice injected with murine melanoma cell lines (reviewed in [100]). The development of retroviral vector delivery systems, such as the RCAS/TVA system, has allowed more rapid assessment of the effects of single or multiple genes on brain metastasis formation. In this model, transgenic mice were engineered to express the avian TVA receptor under the dopachrome tautomerase (*Dct*) promoter, which drives expression of TVA in mitotically active melanocytes. These mice are crossed with strains carrying floxed alleles (*Braf^CA^* for conditional BRAF^V600E^ expression, *Cdkn2a*^*lox*/*lox*^, and Pten^*lox*/*lox*^) to model common human melanoma alterations (*BRAF* mutation plus loss of tumor suppressors *Cdkn2a* and *Pten*). Newborn pups are subcutaneously injected with RCAS viruses encoding Cre recombinase, which induces BRAF^V600E^ expression and deletes *Cdkn2a*/*Pten*, in combination with myrAKT1 or AKT1^E17K^ to enhance PI3K/AKT signaling. This induces spontaneous primary cutaneous melanomas from developmentally normal melanocytes in an intact microenvironment, closely mimicking the human disease. Tumors progress rapidly, with activated AKT1 promoting high metastatic rates, including brain metastases in a substantial fraction of cases (e.g., ~20–80% depending on genetic alterations), enabling studies of metastatic drivers, immune interactions, and therapies in a physiologically relevant, autochthonous setting without injected cell lines (reviewed in [101]). This model was used to identify focal adhesion kinase (FAK) as a critical mediator of melanoma brain metastasis downstream of PI3K/AKT1 signaling [48,102].

## 4. Immunological Perspectives in Melanoma Brain Metastasis

The brain microenvironment is composed of a unique cellular framework, characterized by a distinct immune landscape that differs markedly from other common sites of melanoma metastasis. While the immune landscape of the brain has been historically considered immunologically isolated due to the BBB, recent studies have challenged this paradigm, particularly in the context of brain metastases where the BBB is frequently compromised, permitting infiltration of peripheral immune cells [103]. Thus, in addition to resident microglia, macrophages, and dendritic cells, peripheral immune cells such as neutrophils and lymphocytes are also commonly present within the metastatic brain microenvironment [103].

Tumor-associated macrophages (TAMs) are highly expressed in the brain microenvironment and play a critical role in the pathogenesis of brain metastasis. Two principal TAM subpopulations reside in the CNS: microglia and border-associated macrophages (BAMs). Microglia are phenotypically plastic cells and represent the predominant immune cell population in the healthy brain [104]. Notably, the composition of TAMs is distinctly dynamic and evolves over the course of metastatic progression. In early-stage disease, characterized by smaller metastatic brain lesions, microglia comprise the majority of TAMs. However, as disease progresses and peripheral monocytes infiltrate the brain, the TAM population shifts toward monocyte-derived macrophage cells [105,106,107]. Microglia directly interact with melanoma cells and facilitate their establishment and survival in the brain microenvironment [108,109].

The lymphocytic compartment of the brain microenvironment is primarily composed of T-cells, including both CD4^+^ and CD8^+^ populations, which represent the most phenotypically diverse immune cells in this niche [72,110,111]. Interactions between microglia cells and T-cells have been shown to exert context-dependent effects, contributing to both immunosuppression and immune activation. Several reports demonstrate that microglia-T cell interactions suppress T cell activity and promote metastatic growth [112]. Conversely, brain resident T-cells have also been shown to exhibit antigen-presentation capacity, enhancing anti-tumor immune response within the brain metastatic compartment [113,114].

Despite the challenges of targeting brain metastases, growing insight into the immune microenvironment in the brain, along with recent advances in immunotherapeutic strategies capable of crossing the BBB, offers renewed promise [115]. Immunotherapy has revolutionized the treatment landscape for patients with melanoma. However, baseline immune status plays a critical role in determining therapeutic response. Tumors are often described along an immunologic spectrum ranging from “hot” to “cold” [116]. While primary brain tumors are typically immunologically “cold,” brain metastases, especially originating from melanoma primary tumors, can exhibit “hot” immune profiles due to infiltrating lymphocytes and antitumor immune cells [103]. Because melanoma cells, including metastatic melanoma cells, frequently express high levels of PD-L1, clinical trials have focused on assessing the efficacy of immune checkpoint inhibitors in melanoma brain metastases [72].

A recent Phase II clinical trial evaluating the PD-1 antibody pembrolizumab in patients (both treatment naive and pretreated) from multiple primary tumor types, including melanoma, demonstrated an intracranial response of ~40%. However, more than half of patients experienced treatment-related adverse events, highlighting a major therapeutic obstacle. Similarly, trials evaluating the combination therapy with the CTLA-4 inhibitor ipilimumab and the PD-1 inhibitor nivolumab in melanoma brain metastases reported intracranial responses of 46–57%, yet again with adverse events in over half of all treated patients [23,25]. Thus, recent efforts have focused on enhancing immune activation within the brain microenvironment while overcoming immunosuppressive barriers [117,118]. Encouragingly, studies in other metastatic tumor types, as well as novel immunotherapeutic combination strategies, have demonstrated substantial improvements in intracranial progression-free survival and prolonged time to brain metastasis [71,119,120]. Nonetheless, significant work is needed to improve immunotherapeutic efficacy and tolerability for patients with melanoma brain metastases who have exhausted currently available treatment options.

## 5. Melanoma Brain Metastasis: Unique Challenges and Mechanisms

Brain metastases occur far more frequently than primary brain tumors, and melanoma represents the third most common primary malignancy to metastasize to the brain, following lung and breast cancers [17,121,122,123]. Although lung cancer accounts for the greatest absolute number of brain metastases due to its higher overall incidence, melanoma exhibits the highest relative propensity for CNS dissemination among all solid tumors [17]. Epidemiological analyses have also identified sex-based disparities in melanoma brain metastasis. Recent healthcare database studies demonstrate that male patients have ~22% higher risk of developing brain metastases, and among affected individuals, experience poorer clinical outcomes compared with women [124].

The BBB serves as a formidable obstacle to metastatic dissemination, requiring tumor cells to actively traverse endothelial interfaces to colonize the CNS [125,126]. Although the precise mechanisms remain incompletely defined, emerging evidence suggests that direct interactions between melanoma cells and cerebral endothelial cells facilitate extravasation into the brain parenchyma [127,128]. Advances in in vivo modeling and live-cell imaging have substantially improved our understanding of melanoma brain tropism, highlighting critical points in the metastatic cascade.

Within the CNS microenvironment, astrocytes and microglia represent key stromal populations that critically influence metastatic progression. Astrocytes contribute to BBB integrity through their perivascular end-feet, whereas microglia function as the resident immune cells of the CNS [129,130]. Although these cells were historically viewed as protective against metastatic invasion, accumulating evidence indicates that melanoma cells can co-opt astrocytes and microglia, inducing aberrant activation states that promote tumor survival, invasion, and therapeutic resistance [131,132]. Activated microglia, in particular, have been shown to suppress apoptosis in metastatic melanoma cells, further facilitating intracranial tumor expansion [133]. Collectively, these findings suggest that CNS stromal cells, while initially tumor-suppressive, can be rapidly subverted to support metastatic progression.

In addition to microenvironmental influences, melanoma brain metastases exhibit distinct genomic and transcriptomic profiles compared with primary tumors or extracranial metastases. Specifically, next-generation sequencing has identified increased frequencies of mutations in *BRAF*, *SETD2*, *PBRM1*, and *DICER1*, along with increased PD-L1 expression, underscoring distinct genetic and epigenetic features that may contribute to CNS tropism and therapeutic resistance [134]. Supporting this, Biermann et al. performed multi-modal single-cell and spatial genomics of treatment-naive melanoma brain metastases compared with extracranial metastases and discovered that cancer cells from melanoma brain metastases adopt a ‘neural-like’ cell state, expressing genes with essential roles in synapse formation and interactions among brain niche-cells [84]. This phenotype, beyond baseline neural crest lineage, may enhance brain-metastatic organotropism, invasiveness, and drug resistance, warranting further mechanistic and therapeutic exploration.

## 6. Clinical Management and Multidisciplinary Approaches

Management of melanoma brain metastases requires a comprehensive, multidisciplinary approach involving neuro-oncologists, medical oncologists, radiation oncologists, neurosurgeons, rehabilitation specialists, and palliative care teams, each of whom plays a critical role in optimizing patient outcomes. Given the complexity and heterogeneity of intracranial disease, coordinated care across specialties is essential for effective symptom control, therapeutic decision-making, and longitudinal management.

Neuro-oncologists are central to the care of patients with melanoma brain metastases, as these individuals frequently experience a broad spectrum of neurological complications arising from both tumor burden and treatment-related toxicities [135,136]. Clinical manifestations vary according to lesion location and extent and may include seizures, neurocognitive decline, focal neurological deficits, and altered mental status. Accordingly, neuro-oncologic management emphasizes precise pharmacologic and supportive interventions tailored to individual neurological symptoms, with the overarching goal of preserving function and quality of life [135,136].

Medical oncologists oversee systemic disease management and administration of systemic therapies such as immune checkpoint inhibitors and targeted agents. Although they do not directly perform surgical or radiation-based interventions, medical oncologists play a key role in therapeutic strategy selection, coordination of multidisciplinary care, management of treatment-related adverse effects, and provision of patient education and longitudinal support [137]. Diagnostic radiologists contribute to accurate detection and characterization of brain metastases through advanced imaging techniques, while radiation oncologists determine the appropriateness, modality, and dosing of radiotherapy and manage treatment-related toxicities in close collaboration with the broader care team [138,139]. Despite the substantial symptom burden associated with intracranial disease, palliative care and quality-of-life considerations remain underemphasized in patients with melanoma brain metastases. Patients commonly experience debilitating symptoms, including headaches, seizures, fatigue, and progressive neurological impairment, which significantly disrupt daily functioning. Early integration of palliative care has been shown to improve quality of life and reduce symptom severity, particularly when sustained for longer than three months [140,141]. In addition to addressing physical and psychological distress, palliative care facilitates informed decision-making and aligns treatment strategies with patient goals. Given the rapid and often unpredictable progression of brain metastases, early multidisciplinary involvement, including neurology, psychiatry, oncology, and supportive care services, is essential for optimizing patient-centered outcomes [137,141].

As therapeutic options continue to evolve, there is a growing need for expanded research and education focused on the genetic, molecular, and biological drivers of melanoma metastasis to enable more personalized treatment strategies. Advances in genomic profiling, including liquid biopsy approaches such as CTC analysis and ctDNA detection, now permit minimally invasive acquisition of molecular data that can inform therapeutic selection, monitor disease progression, and detect emerging resistance mechanisms [135].

In parallel, predictive modeling approaches that integrate clinical variables, imaging data, germline genetic markers, and computational analyses are increasingly being used to assess metastatic risk and guide individualized management [27,142,143,144]. These models have demonstrated improved accuracy in early detection and prognostication, supporting more tailored treatment paradigms. Although limitations remain, particularly in model generalizability and data standardization, emerging tools such as liquid biopsy platforms and artificial intelligence-driven radiomics hold significant promise for enhancing metastatic risk prediction and further refining precision care in melanoma [135,145,146].

## 7. Therapeutic Strategies for Melanoma Brain Metastasis

The current standard of care for melanoma brain metastases encompasses three principal therapeutic modalities: systemic therapy (Table 1), surgery, and radiation therapy, delivered either as stereotactic radiosurgery (SRS) or whole-brain radiotherapy (WBRT) [147]. Treatment choice is guided by symptoms, number and size of lesions, tumor profile, and patient performance status [22,148]. In patients with asymptomatic brain metastases and no previous local brain therapy, nivolumab combined with ipilimumab should be considered as first-line therapy, as it has demonstrated an intracranial objective response rate (iORR) of 46%, an intracranial benefit of 57%, and a two-year overall survival (OS) rate of 49%. Extended follow-up suggests this therapy preserves its effectiveness for a minimum of 7 years in patients who have active but asymptomatic brain metastases [23,149]. For patients with BRAF^V600E/K^-mutant disease, combined BRAF and MEK inhibitor therapy is recommended as an alternative first-line option, especially for patients with bulky disease [150]. For patients with symptomatic brain metastases, local therapies such as neurosurgical resection (for large or symptomatic lesions) or SRS should be prioritized to control symptoms and local control, followed by systemic therapy once stabilized [150].

Historically, melanoma brain metastases were associated with a dismal prognosis, with median survival estimates of approximately 4–6 months. However, immunotherapies and advances in radiation delivery have led to meaningful improvements in median survival, which is ~9 months from the time of brain metastasis diagnosis in the modern treatment era [18,151]. Certain historical practices are now considered obsolete, including the routine use of WBRT for patients with limited intracranial disease due to its association with neurotoxicity and cognitive decline without superior efficacy over SRS [152,153], and conventional chemotherapy such as dacarbazine and temozolomide, which demonstrate limited intracranial efficacy despite BBB penetration [152,153,154,155]. Radiation therapy remains a cornerstone of intracranial disease management. SRS delivers high-dose radiation with submillimeter precision to discrete lesions while minimizing exposure to surrounding tissue and is the preferred indication for patients with 1–3 lesions including those with asymptomatic disease who progress on immunotherapy [21,156]. SRS is most commonly administered as a single fraction of 18–24 Gy for lesions ≤ 2 cm, while hypofractionated regimens (e.g., 24–30 Gy in 3–5 fractions) are used for larger lesions or those located near critical brain structures to reduce the risk of radiation-induced necrosis [157]. In contrast, WBRT exposes the entire brain to radiation and is generally reserved for patients with extensive intracranial disease, leptomeningeal involvement, or those who are not candidates for surgery or SRS. When employed, WBRT is typically delivered as 30 Gy in 10 fractions, with dose adjustments and hippocampal-sparing techniques increasingly utilized to mitigate neurocognitive toxicity [158]. Radiotherapy may be introduced first for symptomatic lesions, following surgical resection as adjuvant therapy, or at progression during systemic treatment. Comparative studies have demonstrated no significant difference in recurrence rates between SRS and WBRT, with a trend toward improved survival in patients treated with SRS [159]. The most favorable outcomes are observed in patients undergoing surgical resection followed by adjuvant SRS [21].

Systemic therapy for brain metastases consists of immunotherapy and molecularly targeted agents (Table 1). Historically, systemic therapy alone has demonstrated limited efficacy against brain metastases, likely due to restricted drug penetration across the BBB, while radiotherapy alone offers only marginal benefit over supportive care and is frequently associated with neurotoxicity and subsequent cognitive decline [160,161]. In 2022, joint American Society of Clinical Oncology (ASCO), Society for Neuro-Oncology (SNO), and American Society for Radiation Oncology (ASTRO) guidelines recommended combination immunotherapy with ipilimumab plus nivolumab for patients with asymptomatic melanoma brain metastases, irrespective of *BRAF* mutation status [150].

Multiple studies have evaluated mutation-specific targeted therapies in melanoma brain metastases. In the Phase II BREAK-MB trial, treatment with dabrafenib achieved an overall intracranial response rate of 35% in patients with *BRAF^V600E/K^* alterations [162]. New evidence also supports the efficacy of newer BRAF and MEK inhibitors, including recently approved encorafenib and binimetinib for patients with brain metastatic melanoma [163,164,165]. Other targeted approaches have yielded mixed results. For example, the PI3K inhibitor buparlisib was well tolerated but produced no intracranial responses [20], while a Phase II study of the CDK4/6 inhibitor abemaciclib failed to meet its primary endpoint despite evidence of BBB penetration and intracranial benefit in a subset of patients [166]. Despite these advances, effective pharmacologic treatment of melanoma brain metastases remains limited by the BBB, which is composed of tightly connected endothelial cells, pericytes, and astrocytic end-feet that restrict CNS entry [167]. More favorable outcomes have been observed in patients undergoing surgical resection followed by immunotherapy, although immune-related adverse events (IRAEs) limit treatment durability [168].

Recent trials have highlighted the potential benefits of neoadjuvant immunotherapy for patients with locally advanced melanoma with clinically involved regional lymph nodes. Studies comparing neoadjuvant vs. adjuvant pembrolizumab in resectable late-stage cutaneous melanoma demonstrated improved outcomes with neoadjuvant treatment; however, these trials excluded patients with brain metastases, underscoring the continued therapeutic gap for unresectable intracranial disease [169].

Therapeutic resistance remains a major clinical challenge in advanced melanoma. A key mechanism of therapeutic resistance arises from tumor-stromal interactions [19]. Melanoma cells form gap junctions with astrocytes via connexin-43, which facilitates the settlement of melanoma cells in the brain [170]. Emerging therapeutic strategies aim to overcome resistance and exploit CNS-specific vulnerabilities. Chimeric antigen receptor (CAR) T cells have demonstrated efficacy in hematologic malignancies and show promise in melanoma [171,172,173,174,175]. Oncolytic viruses represent another promising approach, leveraging tumor-selective viral replication to induce direct oncolysis and stimulate anti-tumor immunity [176]. A Phase III clinical trial led to FDA-approval of Talimogene Laherparepvec (TVEC), a modified herpes simplex virus type 1 encoding granulocyte-macrophage colony-stimulating factor for advanced melanoma [177] (Table 1). However, BBB penetration remains an obstacle to oncolytic virus efficacy, and further studies are needed to assess CNS delivery of candidate viruses such as parvovirus H-1PV [178]. Additional innovative approaches include repurposing BBB-penetrant anti-psychotic agents such as trifluoperazine, fluphenazine, and clozapine, which disrupt lysosomes, induce cell-cycle arrest, and modulate the immune microenvironment in melanoma brain metastases [179,180]. While targeting RAS was long considered unfeasible, the advent of novel RAS inhibitors has recently revolutionized treatment for cancers driven by oncogenic RAS signaling and is now being clinically explored in NRAS-mutant melanoma [181,182], building on earlier evidence of activity with MEK inhibitors in this subgroup [183].

Almazan and colleagues demonstrated that combined inhibition of FAK and RAF/MEK signaling suppressed melanoma growth, reduced brain metastasis formation, and prolonged survival in vivo. FAK inhibition alone significantly reduced the development of brain metastases, while the combined inhibition of FAK and RAF/MEK induced tumor regression leading to prolonged survival in preclinical models [102]. Notably, this study employed the novel RAF/MEK inhibitor, avutometinib, which functions as a molecular clamp to inhibit MAPK signaling. Promising preclinical studies such as these have catalyzed ongoing clinical trials for solid tumors using these combination therapies and offered renewed hope for patients with melanoma brain metastases [184,185] (Table 2).

## 8. Future Directions and Research Gaps

Future research on melanoma brain metastases converges on a few major challenges, including overcoming therapeutic barriers to drug delivery across the BBB, advancing biomarker identification to detect early metastatic spread, and further elucidating mechanisms of how melanoma spreads to the brain. A major priority of metastatic melanoma research is to identify therapeutics that can reach the brain microenvironment, as the BBB prevents adequate drug penetration and can lead to uneven drug distribution [125]. This, along with unique immune profiles and an immunosuppressive microenvironment in the brain, makes current systemic treatments for melanoma brain metastases less effective [186]. Concurrent efforts in biomarker discovery aim to identify molecular signatures to help predict patients with a higher risk of brain metastasis in order to guide therapeutic options. Multi-omics platforms (such as genomic, transcriptomic, proteomic, and spatial profiling approaches) and liquid biopsies are leveraged to uncover new predictive biomarkers and therapeutic targets [187].

Equally important is expanding our mechanistic understanding of how melanoma cells invade and adapt in the brain microenvironment, from reaching the BBB to co-opting astrocytes and microglia to evade immune surveillance and ultimately propagate in the brain. Emerging approaches such as multi-omic profiling and AI-driven modeling hold promise for accelerating progress. For example, a recent multi-omic analysis revealed distinct immunosuppressive and metabolic tumor microenvironments in melanoma brain metastases [85]. Moreover, machine learning models can integrate radiomic, genomic, and clinical data in order to improve prognostic predictions and aid in personalizing treatment [188]. By addressing these gaps to improve drug delivery across the BBB, establish robust biomarkers for early metastatic spread, and further identifying brain colonization, the field will continue to make strides to deliver more effective and personalized interventions for patients with melanoma brain metastases.

## 9. Conclusions

Melanoma brain metastasis represents one of the most aggressive and therapeutically challenging manifestations of metastatic cancer, driven by both tumor-intrinsic programs and the unique constraints of the CNS [1,17]. Advances in molecular biology, multi-omic profiling, and preclinical modeling have clarified mechanisms of CNS tropism, including lineage-specific transcriptional programs, metabolic rewiring, and stromal co-option by astrocytes and microglia [41,108,131,189,190,191]. However, these insights are only the beginning in translating effective intracranial therapies. Despite meaningful advances in immune checkpoint blockade and targeted MAPK inhibition [23,49,67,68,102,165,168,169,192,193,194], durable intracranial response remains limited by intratumoral heterogeneity and adaptive resistance pathways [85]. Looking forward, future studies should prioritize the development of CNS-penetrant therapies and rational combination strategies. Integration of liquid biopsy platforms, advanced imaging, multi-omic profiling, coupled with AI-driven predictive modeling, offers a promising framework to detect micrometastases and guide treatment strategies. By aligning mechanistic discovery with intracranial-focused clinical trial design, this review provides a foundation for future translational and clinical efforts aimed at improving survival in patients with melanoma brain metastases.

## 10. Materials and Methods

This narrative review synthesizes preclinical, translational, and clinical evidence on the molecular mechanisms, immunological landscape, and therapeutic strategies for melanoma brain metastasis. Literature was identified through targeted searches of PubMed, Scopus, Web of Science, and Embase using combinations of terms such as “melanoma”, “brain metastasis,” “NRAS,” “BRAF,” “PI3K/AKT,” “immune checkpoint inhibitors,” “brain microenvironment,” “preclinical models,” and “therapeutic resistance” (with Boolean operators AND/OR as appropriate). Reference lists of key articles and prior reviews were hand-searched for additional sources, and forward citation tracking via Google Scholar was employed to capture emerging studies. Publications were included if they were peer-reviewed original research, clinical trials, systematic reviews, or guidelines in English, directly addressing melanoma brain metastasis biology, models, or treatments; editorials, conference abstracts, non-human-only studies without translational relevance, and non-melanoma-specific brain metastasis papers were excluded. Approximately 500 articles were screened based on title and abstracts for relevance, with 195 selected for inclusion due to their seminal contributions, methodological rigor, representation of diverse perspectives (e.g., preclinical vs. clinical, targeted vs. immunotherapy approaches), and coverage of multidisciplinary aspects. Synthesis followed an integrative narrative approach, organizing findings thematically by mechanisms (e.g., genetic drivers, EMT-like plasticity, tumor microenvironment), models, therapies, and future directions, with critical appraisal of study strengths, limitations, and implications for rigor and reproducibility. The narrative format was chosen for its suitability in interpreting a broad, heterogeneous field where quantitative meta-analysis is not feasible due to variability in study design and endpoints.

## Figures and Tables

**Table 1:** Summary of FDA approved treatments for patients with unresectable or metastatic melanoma [5].

Treatment	Medication	Indication	Dose	Route	Schedule	Duration
Immunotherapy	Pembrolizumab	Unresectable/MM	200 mg or 400 mg	IV	Every 3 wks (200 mg) or 6 wks (400 mg)	Until progression or unacceptable toxicity
Immunotherapy	Nivolumab	Unresectable/MM	240 mg or 480 mg (≥40 kg); 3 mg/kg or 6 mg/kg (<40 kg)	IV	Every 2 wks (240 mg) or 4 wks (480 mg)	Until progression or unacceptable toxicity
Immunotherapy	Nivolumab + Ipilimumab	Unresectable/MM	Nivolumab 1 mg/kg + Ipilimumab 3 mg/kg, then Nivolumab 240 mg	IV	Ipi/Nivo every 3 wks (4 doses) then Nivolumab every 2 wks	Until progression or unacceptable toxicity
Immunotherapy	Nivolumab + Relatlimab	Unresectable/MM	480 mg Nivolumab + 160 mg Relatlimab	IV	Every 4 wks	Until progression or unacceptable toxicity
Immunotherapy	Talimogene laherparepvec (T-VEC)	Unresectable with injectable lesions	Initial dose: ≤4 mL of 10^6^ PFU/mL	Intralesional	Initial dose day 1; 2nd dose at wk 3; then every 2 wks	Until progression or unacceptable toxicity
Immunotherapy	Tebentafusp	HLA-A*02:01+ unresectable or metastatic uveal melanoma	20 mcg (Day 1), 30 mcg (Day 8), 68 mcg (Day 15 and weekly thereafter)	IV	Weekly after initial ramp-up	Until progression or unacceptable toxicity
Targeted Therapy	Dabrafenib + Trametinib	BRAF^V600E/K^-unresectable/MM	Dabrafenib 150 mg + Trametinib 2 mg	Oral	Dabrafenib twice daily, Trametinib once daily	Until progression or unacceptable toxicity
Targeted Therapy	Vemurafenib + Cobimetinib	BRAF^V600E/K^-Unresectable/MM	Vemurafenib 960 mg (Days 1–21) then 720 mg; cobimetinib 60 mg	Oral	Vemurafenib twice daily, Cobimetinib once daily for 21 days on/7 days off	Until progression or unacceptable toxicity
Targeted Therapy	Encorafenib + Binimetinib	BRAF^V600E/K^-Unresectable/MM	Encorafenib 450 mg + Binimetinib 45 mg	Oral	Encorafenib once daily, Binimetinib twice daily	Until progression or unacceptable toxicity
Combination (Immuno + Targeted)	Atezolizumab + Cobimetinib + Vemurafenib	BRAF^V600^-Unresectable/MM	Atezolizumab 840 mg; Cobimetinib 60 mg; Vemurafenib 960 mg (Days 1–21) then 720 mg	Atezolizumab IV; others oral	Atezolizumab every 2 weeks; Cobimetinib 21 on/7 off; Vemurafenib twice daily	Until progression or unacceptable toxicity (initial 28-day cycle without atezolizumab)
Cellular Therapy	Lifileucel	Unresectable/MM (post-PD-1 and BRAF/MEK)	7.5–72 × 10^9^ viable cells	IV single infusion	One-time	Single dose, following lymphodepletion and IL-2 administration

MM: metastatic melanoma; IV: intravenous; wks: weeks; wk: week.

**Table 2:** Summary of active clinical trials for patients with melanoma brain metastases ^1^.

NCT Number	Phase	Primary Outcome	Title
NCT06194929	I/II	ORR	Defactinib and Avutometinib, With or Without Encorafenib, for the Treatment of Patients With Brain Metastases From Cutaneous Melanoma (DETERMINE)
NCT03563729	II	6 month PFS	Melanoma Metastasized to the Brain and Steroids (MEMBRAINS)
NCT06163820	I/II	S&T, iORR	Bevacizumab and ICIs + hSRT in Symptomatic Melanoma Brain Metastases (BETTER)
NCT06712927	II	iORR	Trial of Relatlimab, Nivolumab, and Ipilimumab in Patients With Asymptomatic and Symptomatic Melanoma Brain Metastases
NCT05704647	II	iORR, AEs	Phase II Study of Nivolumab in Combination With Relatlimab in Patients With Active Melanoma Brain Metastases
NCT06887088	II	icPFS	Encorafenib and biNimetinib Followed by CEmiplimab and FiAnLimab in Patients With BRAF Mutant melanOma and Symptomatic Brain Metastases (ENCEFALO)
NCT04511013	II	PFS	A Study to Compare the Administration of Encorafenib + Binimetinib + Nivolumab Versus Ipilimumab + Nivolumab in BRAF-V600 Mutant Melanoma With Brain Metastases
NCT03340129	II	Neurologic death	Anti-PD 1 Brain Collaboration + Radiotherapy Extension (ABC-X Study)
NCT05026983	II	PFS, DLTs, and AEs	Binimetinib and Encorafenib for the Treatment of Metastatic Melanoma and Central Nervous System Metastases
NCT04899908	II	Local Recurrence	Stereotactic Brain-directed Radiation With or Without Aguix Gadolinium-Based Nanoparticles in Brain Metastases
NCT05703269	III	Occurrence of ARE	Comparing Single vs. Multiple Dose Radiation for Cancer Patients With Brain Metastasis and Receiving Immunotherapy (HYPOGRYPHE)
NCT05909618	I/II	S&T, ORR	Crizanlizumab Alone or in Combination With Nivolumab for Glioblastoma and Melanoma With Brain Metastases
NCT05522660	III	CNS-specific PFS	Immunotherapy or Targeted Therapy With or Without Stereotactic Radiosurgery for Patients With Brain Metastases From Melanoma or Non-small Cell Lung Cancer (USZ-STRIKE)
NCT05588453	I/II	DLTs, iORR	Natural Killer Cell Therapy (UD TGFbetai NK Cells) and Temozolomide for the Treatment of Stage IV Melanoma Metastatic to the Brain
NCT06500455	III	Time to local failure	Testing Longer Duration Radiation Therapy Versus the Usual Radiation Therapy in Patients With Cancer That Has Spread to the Brain
NCT04021420	I/II	MSD	Safety and Efficacy of Sonocloud Device Combined With Nivolumab in Brain Metastases From Patients With Melanoma (SONIMEL01)
NCT06346067	III	PFS and OS	A Study to Assess Naporafenib (ERAS-254) Administered With Trametinib in Patients With NRAS-mutant Melanoma (SEACRAFT-2)
NCT01503827	III	Intracranial failure	Whole Brain Radiotherapy Following Local Treatment of Intracranial Metastases of Melanoma (WBRTMel)
NCT02460068	III	OS	A Study of Fotemustine (FTM) vs. FTM and Ipilimumab (IPI) or IPI and Nivolumab in Melanoma Brain Metastasis (NIBIT-M2)

^1^ Trials focus on melanoma brain metastasis or do not exclude patients with melanoma brain metastasis. AE: adverse events; ARE: Adverse Radiation Effect; CNS: central nervous system; DLTs: dose limiting toxicities; icPFS: intracranial progression-free survival; iORR: intracranial objective response rate; MSD: Most Successful Dose; ORR: objective response rate; OS: overall survival; PFS: progression-free survival; S&T: Safety and tolerability (source: clinicaltrials.gov).

## Data Availability

No new data were created or analyzed in this study. Data sharing is not applicable to this article.

## Abbreviations

The following abbreviations are used in this manuscript:

18F-FDG	18F-fluorodeoxyglucose
AE	Adverse events
ARE	Adverse Radiation Effect
AKT	Serine/threonine kinase
APE1	Apurinic/apyrimidinic endodeoxyribonuclease 1
ASCO	American Society of Clinical Oncology
ASTRO	American Society for Radiation Oncology
BAMs	Border-associated macrophages
BBB	Blood–brain barrier
BRAF	B-Raf proto-oncogene
CAR	Chimeric antigen receptor
CDK4/6	Cyclin-dependent kinase 4/6
CMCs	Circulating melanoma cells
CNS	Central nervous system
CSF1R	Colony-stimulating factor 1 receptor
CTCs	Circulating tumor cells
CTL	Cytotoxic T lymphocyte
CTLA-4	Cytotoxic T-lymphocyte antigen 4
ctDNA	Circulating tumor DNA
DICER1	Dicer 1, ribonuclease III
DNA	Deoxyribonucleic acid
DLTs	Dose limiting toxicities
ECM	Extracellular matrix
EMT	Epithelial-to-mesenchymal transition
ERK	Extracellular signal-regulated kinase
FAK	Focal adhesion kinase
FATP2	Fatty acid transport protein 2
FDG-PET	Fluorodeoxyglucose positron emission tomography
FET	O-(2-18F-Fluoroethyl)-l-tyrosine
GEMM	Genetically-engineered mouse model
GTPase	Guanosine triphosphatase
ICIs	Immune checkpoint inhibitors
icPFS	Intracranial progression-free survival
IFNβ	Interferon beta
IL-8	Interleukin 8
IL-10	Interleukin 10
iORR	Intracranial objective response rate
IRAEs	Immune-related adverse events
IV	Intravenous
MAPK	Mitogen-activated protein kinase
MEK	Mitogen-activated protein kinase
MITF	Microphthalmia-associated transcription factor
MRI	Magnetic resonance imaging
MSD	Most Successful Dose
NGFR	Nerve growth factor receptor
NIH	National Institutes of Health
NRAS	Neuroblastoma RAS viral oncogene
ORR	Objective response rate
OS	overall survival
PBRM1	Polybromo 1
PD-1	Programmed cell death protein 1
PD-L1	Programmed death-ligand 1
PDGFR	Platelet-derived growth factor receptor
PDX	Patient-derived xenograft
PET	Positron emission tomography
PFS	progression-free survival
PI3K	Phosphatidylinositol 3-kinase
PRRX1	Paired related homeobox 1
PTEN	Phosphatase and tensin homolog
RNA	Ribonucleic acid
SETD2	SET domain containing 2
sFRP2	Secreted frizzled related protein 2
SLC27A2	Solute carrier family 27 member 2
SNO	Society for Neuro-Oncology
SOX10	SRY-box transcription factor 10
SPARC	Secreted protein acidic and rich in cysteine
SRS	Stereotactic radiosurgery
S&T	Safety and tolerability
TAMs	Tumor-associated macrophages
TCF/LEF	Transcription factor/lymphoid enhancer-binding factor
TFs	Transcription factors
TGF-β	Transforming growth factor beta
TME	Tumor microenvironment
TVEC	Talimogene Laherparepvec
UV	Ultraviolet
WBRT	Whole-brain radiotherapy
Wks	Weeks
YAP1	Yes-associated protein 1
ZEB1	Zinc finger E-box binding homeobox 1
ZEB2/SNAIL2	Zinc finger E-box binding homeobox 2/snail family transcriptional repressor 2
